# Survey of oxaliplatin-associated neurotoxicity using an interview-based questionnaire in patients with metastatic colorectal cancer

**DOI:** 10.1186/1471-2407-5-116

**Published:** 2005-09-16

**Authors:** Gregory D Leonard, Maurice A Wright, Mary G Quinn, Suzanne Fioravanti, Nancy Harold, Barbara Schuler, Rebecca R Thomas, Jean L Grem

**Affiliations:** 1 Cancer Therapeutics Branch, Center for Cancer Research, National Cancer Institute-Navy Medical Oncology Program, National Naval Medical Center, Bethesda, MD 20889-5105, USA; 2 Medical Oncology Research Unit, Center for Cancer Research, National Cancer Institute-Navy Medical Oncology Program, National Naval Medical Center, Bethesda, MD 20889-5105 USA

## Abstract

**Background:**

New chemotherapy regimens for patients with colorectal cancer have improved survival, but at the cost of clinical toxicity. Oxaliplatin, an agent used in first-line therapy for metastatic colorectal cancer, causes acute and chronic neurotoxicity. This study was performed to carefully assess the incidence, type and duration of oxaliplatin neurotoxicity.

**Methods:**

A detailed questionnaire was completed after each chemotherapy cycle for patients with metastatic colorectal cancer enrolled in a phase I trial of oxaliplatin and capecitabine. An oxaliplatin specific neurotoxicity scale was used to grade toxicity.

**Results:**

Eighty-six adult patients with colorectal cancer were evaluated. Acute neuropathy symptoms included voice changes, visual alterations, pharyngo-laryngeal dysesthesia (lack of awareness of breathing); peri-oral or oral numbness, pain and symptoms due to muscle contraction (spasm, cramps, tremors). When the worst neurotoxicity per patient was considered, grade 1/2/3/4 dysesthesias and paresthesias were seen in 71/12/5/0 and 66/20/7/1 percent of patients. By cycles 3, 6, 9, and 12, oxaliplatin dose reduction or discontinuation was needed in 2.7%, 20%, 37.5% and 62.5% of patients.

**Conclusion:**

Oxaliplatin-associated acute neuropathy causes a variety of distressing, but transient, symptoms due to peripheral sensory and motor nerve hyperexcitability. Chronic neuropathy may be debilitating and often necessitates dose reductions or discontinuation of oxaliplatin. Patients should be warned of the possible spectrum of symptoms and re-assured about the transient nature of acute neurotoxicity. Ongoing studies are addressing the treatment and prophylaxis of oxaliplatin neurotoxicity.

## Background

Colorectal cancer is the third commonest cause of non-skin cancers in men and in women with a total of 146,940 new cases and 56,730 deaths estimated in the United States in 2004 [1]. Approximately 25% of patients present with metastatic disease. Despite improvements in adjuvant therapy, a substantial number of patients with localized disease will ultimately develop metastatic disease. The 5-year survival for patients with metastatic colorectal cancer is less than 10%. The role of chemotherapy in patients with unresectable disease as a neoadjuvant approach to make resection possible is currently being explored in clinical trials; retrospective analyses suggest that patients who can undergo complete surgical resection of hepatic metastases after neoadjuvant chemotherapy may have a survival approaching 50% [2].

The standard chemotherapy regimen used as first-line treatment of colorectal cancer for many years was bolus 5-FU modulated by (LV), but this has recently been superseded by newer combination regimens involving LV-modulated 5-FU given as a mixed bolus and continuous infusion with the addition of either oxaliplatin or irinotecan. Oxaliplatin, an organoplatinum complex, produces cytotoxicity via the formation of DNA adducts which interfere with DNA replication and transcription and lead to induction of apoptosis. The different spectrum of clinical activity and toxicity of oxaliplatin compared to cisplatin and carboplatin is believed to be due to its 1,2-diaminocyclohexane structure, which forms bulkier DNA adducts. A Phase III trial in patients with metastatic colorectal cancer comparing oxaliplatin and biweekly LV-modulated mixed bolus and infusional 5-FU (FOLFOX 4) to bolus 5-FU/LV given daily for five days every month demonstrated superiority in both response rate and progression-free survival [3]. Another Phase III trial demonstrated a significant survival advantage with FOLFOX-4 compared to irinotecan and bolus 5-FU/LV (median survival 19.5 vs 15 months, p = 0.0001) [4]. A randomized trial comparing sequential administration of biweekly LV-modulated mixed bolus and infusional 5-FU combined with either oxaliplatin (FOLFOX 4) or irinotecan (FOLFIRI) followed by the other regimen at the time of disease progression showed equivalent survival of about 21 months for both sequences [5]. The Food and Drug Administration approved FOLFOX-4 as salvage therapy of metastatic colorectal cancer in August, 2002, and as first-line therapy in January, 2004. Oxaliplatin has also shown activity in other platinum-sensitive tumors, including in some cancer patients with documented cisplatin-refractory disease [6-8].

The dose-limiting toxicity of oxaliplatin is neurotoxicity [9,10]. The neurotoxicity has two distinct manifestations: acute, transient symptoms are due to peripheral sensory and motor neuron hypersensitivity; cumulative, persistent symptoms are due to chronic peripheral sensory neuropathy [9-12]. The pathogenesis of the acute neuropathy is believed to be due to a channelopathy (a temporary dysfunction of an ion channel in the nerve membrane), while chronic sensory neuropathy may be a result of direct, cumulative neurotoxic effects resulting from platinum accumulation in the dorsal root ganglion [11-15]. A number of grading systems have been used to report oxaliplatin-associated neurotoxicity. Clinical studies involving oxaliplatin appear to have largely described the chronic sensory neuropathy, whereas information on the acute peripheral motor and sensory neuropathy is more limited. In order to obtain information on the incidence and type of oxaliplatin-associated acute and chronic neurotoxicity, we devised a questionnaire to be used in a phase I study of oxaliplatin and capecitabine

## Methods

### Voluntary subjects

Patients were enrolled in a Phase I study examining the safety and feasibility of administering a fixed dose of oxaliplatin with escalating doses of capecitabine to patients with advanced colorectal or small bowel adenocarcinoma. The protocol was approved by the Cancer Therapy Evaluation Program, NCI, and Institutional Review Boards of the NCI and the National Naval Medical Center. All patients gave written informed consent. As part of the trial, we collected information on neurotoxicity experienced by the patients during therapy. We previously reported information obtained using EMG and NCS as an objective assessment of the neurotoxicity [11,12]. The objective of the present analysis was to determine the incidence, type and duration of neurotoxicity using a questionnaire that was filled in by a research nurse during an interview with the subject.

### Chemotherapy and dose modifications

Oxaliplatin 130 mg/m^2 ^was infused by vein over 2-hours on day one, while a total daily dose of capecitabine ranging from 1,200 to 3,000 mg/m^2 ^was given in two divided doses on days 1–5 and 8–12 of a planned 21-day cycle. As part of this intramural NCI research study, patients were hospitalized overnight at no charge following the first infusion of oxaliplatin to allow monitoring for acute neurotoxicity, and patients were interviewed the next morning to record any acute symptoms. Protocol therapy was continued until evidence of disease progression occurred, unacceptable toxicity developed, or the patient elected to withdraw. A neurotoxicity grading system developed by the pharmaceutical sponsor was used to describe the paresthesias/dysesthesias, which could be cold-induced or not: grade 1, symptoms of short duration that resolve and do not interfere with function; grade 2, interfering with some functions, but not with the basic activities of daily living; grade 3, pain or functional impairment that interfere with activities of daily living; grade 4, persistent symptoms that are disabling or life-threatening.

If subjects experienced acute, transient neurotoxicity that was particularly bothersome, the duration of infusion was increased to six hours in subsequent cycles. The dose of oxaliplatin was decreased by 25% if grade 2 neurotoxicity persisted between cycles. The dose of oxaliplatin was decreased by 25% for grade 3 neurotoxicity that resolved before the next cycle of therapy, while the drug was discontinued for either grade 3 neurotoxicity that persisted between cycles, or for grade 4 neurotoxicity, regardless of duration. In a subset of twelve patients, an empiric trial of carbamazepine was begun five days prior to the second dose of oxaliplatin and continuing for a total of seven days as previously reported to assess any possible impact on acute neurotoxicity symptoms and neurophysiologic studies [11].

### Neurologic assessment

Subjects were asked to keep a daily diary of side-effects. A research nurse interviewed the subjects and filled out a questionnaire that characterized the incidence and type of neurotoxicity symptoms and how long they lasted. The questionnaire was divided into 3 parts addressing the upper extremity, the lower extremity and orofacial area (Tables 1, 2, 3), and was completed after each 3-week cycle of chemotherapy. Objective neurological assessment was also obtained by physical exam in all patients at the start of each cycle. The results of serial EMG and NCS tests in a subset of patients have been reported previously [12].

## Results

### Demographics

Eighty-six patients (56 males/30 females) were included in this assessment; 69 were Caucasian, 14 were African-American, and three were Hispanic. The median age was 56 years (range 26–74). The location of the primary tumor was colon (76 patients), rectum (8) or appendix (2). The median ECOG performance status was 1 (range 0–2). The majority of the patients had undergone prior surgical resection of their primary tumor; 90% had received prior 5-FU, and two-thirds had received prior irinotecan-based therapy.

### Use and analysis of the questionnaire

The questionnaire was a tool that was designed to help capture the variety and location of potential neurotoxic side effects that the patients might experience. Patients were specifically queried about symptoms occurring the upper extremities, lower extremities, and face/mouth. Patients were asked if they had each of the specific symptoms, yes or no. If yes, then there were two parts to the assessment: first, whether the patient felt the symptoms were minimal to very prominent; second, whether the symptoms affected the daily activities hardly at all through a range that the subject was extremely bothered by the symptoms in a functional sense. The following scores on the questionnaire corresponded to the following neurotoxicity grades: 1,2 = grade 1; 3 = grade 2; 4 = grade 3; 5 = grade 4.

### Neuropathy

Dysesthesia, herein defined as painful or distressing sensations experienced in the absence of stimulation, was experienced by 87.2% of patients. Paresthesia, herein defined as non-painful but abnormal sensations such as numbness or tingling, was experienced by 94.3% of patients at some point during oxaliplatin-based chemotherapy (Table 4). For most patients, the worst grade of dysesthesia and paresthesia was grade 1 (70.9% and 66.3% respectively). However, some patients experienced functional impairment affecting activities of daily living. One patient developed an ascending sensorimotor syndrome after the fourth dose of oxaliplatin that led to profound weakness in the upper and lower extremities without respiratory impairment, although it was uncertain whether this was due to oxaliplatin or due to the development of an unrelated neurological condition [16].

**Figure 1:**
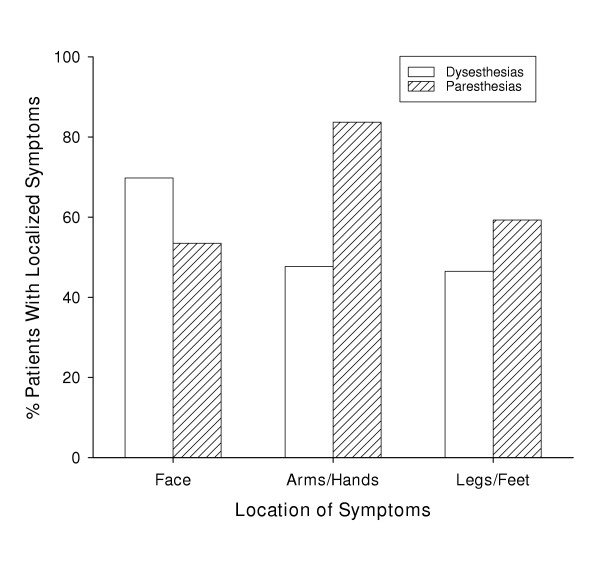
Location of dysesthesias/paresthesias during therapy with oxaliplatin and capecitabine (n = 86 subjects).

Dysesthesias occurred more often in the face than in the extremities, perhaps due to acute, painful cold sensitivity experienced while eating, drinking or handling a cold object (Figure 1). Paresthesias occurred more often in the hands and feet, which may be consistent with the glove and stocking distribution associated with chronic peripheral neuropathies.

**Figure 2:**
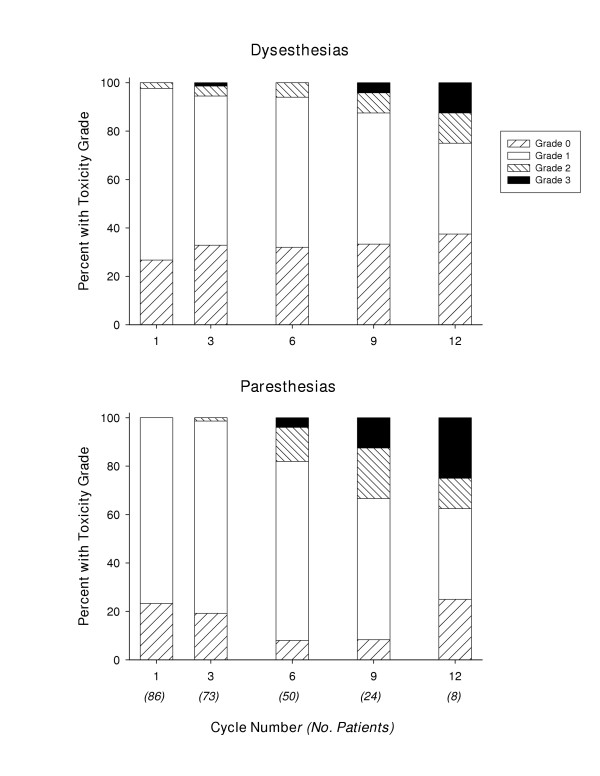
Percentage of subjects with dysesthesias/paresthesias as a function of number of cycles. The number of subjects at each cycle is shown in parentheses.

The duration of the neuropathy increased as the cumulative exposure to oxaliplatin increased (Table 5). After the first cycle of chemotherapy, the median duration of dysesthesia was only 5 days, whereas it was 21 days in patients who received 12 cycles of chemotherapy. The median duration of paresthesia after cycle one was 7 days, but after cycle 12 was 21 days or longer. The proportion of patients who complained of dysesthesia of any grade after one cycle of chemotherapy (73%) was similar to the proportion of patients who complained of paresthesia (77%) (Figure 2). After six cycles of chemotherapy, 68% of patients complained of dysesthesia, whereas 92% of patients complained of paresthesia. The increased incidence of paresthesia is consistent with the development of a cumulative sensory neuropathy in most patients. The relatively stable incidence of dysesthesia may reflect that such symptoms are more commonly associated with the transient hyperexcitability phenomenon. The acute symptoms do not appear to be cumulative phenomenon. By cycles 9 and 12, the incidence of grade 3 dysesthesia and paresthesia became noticeable. Remarkably, some patients did not complain of any paresthesia or dysesthesia even after 12 cycles of therapy. By cycles three and six, oxaliplatin dose reductions for neurotoxicity were needed in 2.7% and 20% of patients. By cycle 9, 37.5% of patients required a dose reduction (4% of these had discontinued oxaliplatin); by cycle 12, 62.5% of remaining patients had discontinued oxaliplatin.

**Figure 3:**
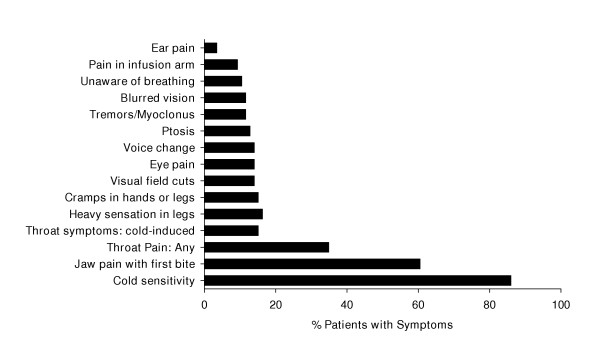
Percentage of subjects experiencing specific types of symptoms due to acute peripheral sensory and motor nerve hyperexcitability (n = 86 subjects).

Acute neurotoxicity symptoms were recorded with particular focus on the nature of the symptoms. The commonest symptom experienced was cold sensitivity (>80%), which was often reported to be painful by patients (Figure 3). Pain with the initial bite of food was noted by about 60% of the patients, most likely attributed to masticatory spasm. Some symptoms were of very short duration (lasting only seconds or at most a few minutes), while other lasted for several hours (ptosis, sensation of leg heaviness); both short- and longer-lived symptoms could be of major concern to both patient and physician. The most distressing symptom was pharyngolaryngeal dysesthesia, which patients often described as a poor awareness that they were breathing. Other patients noted transient hoarseness or change in their voice, and some commented on visual field deficits or ocular pain. Despite the fact that symptoms resolved spontaneously over a short period of time, many patients required anxiolytics to reduce their level of distress. A subset of patients who received carbamazepine as a prophylactic measure to reduce the symptoms of acute neuropathy did not appear to derive any benefit from this approach [12].

## Discussion

Oxaliplatin is an integral component of the various FOLFOX regimens, which have become a standard treatment for metastatic and node-positive colorectal cancer [3,4,17]. Encouraging results have been reported from phase II trials using oxaliplatin in combination with pemetrexed, raltitrexed or capecitabine in patients with metastatic colorectal cancer [18-24]. Enthusiasm regarding the efficacy of oxaliplatin/5-FU/LV regimens has been tempered by the associated neurotoxicity which can significantly impair the quality of life of patients and limits the optimal use of oxaliplatin. Many published trials provide limited details concerning the precise type and location of neurotoxicities experienced, and most appear to have focused on the cumulative sensory neuropathy. Data on the reported incidence of neuropathy from randomized trials, presented in Table 6, may be influenced by the toxicity scale employed, the cumulative oxaliplatin dose, and schedule of administration (3–5,1 7, 25–29). For three trials that employed 85 mg/m^2 ^oxaliplatin given over 2 hours as first-line therapy for metastatic colorectal cancer, the incidence of grade 3 or worse neuropathy was reported to be a mean of 15.4%. A higher incidence of grade 3 neuropathy (31–34%) was reported when oxaliplatin was given at 20 mg/m^2 ^as a 24-hour infusion daily for 5 days every 3 weeks or as 100 mg/m^2 ^over 2 hours every 2 weeks. The incidence of grade 3 neuropathy was lower with IROX, in which 85 mg/m^2 ^is given over 2 hours every 3 weeks. The lowest incidence of serious neurotoxicity has been reported in trials in which FOLFOX is used as second- or third-line salvage therapy, presumably because the cumulative dose of oxaliplatin is less than when employed as first-line therapy.

Although the symptoms associated with acute peripheral sensory and motor nerve hyperexcitability seem to be transient in nature, they can cause distressing symptoms in patients. We used a questionnaire that was filled out by the research nurse during a face- to face interview with the patient. All patients kept a daily calendar diary of side effects, and were interviewed the morning after the initial dose of oxaliplatin. This approach provided detailed information on the incidence, type, location and duration of neuropathy experienced by patients. Since all research subjects were admitted for their first cycle of chemotherapy, close observation for acute neurological symptoms and next-day interviews with patients was possible. These measures may have facilitated identification of symptoms or signs that perhaps would otherwise have gone unreported.

Our study demonstrated that most patients experience either dysesthesia or paresthesia at some point during their treatment, although the worst toxicity grade was grade 1 in most patients. Due to stringent dose modification guidelines, only a small proportion of patients experience serious functional impairment. However, as the cumulative dose of oxaliplatin increases, patients are more likely to experience paresthesia due to a cumulative sensory neuropathy. In patients who receive more than 6 cycles of chemotherapy (projected maximum cumulative dose on this every 3-week schedule of 780 mg/m^2^), the neuropathy can become persistent and affect the subject's ability to perform routine activities of daily living. Oxaliplatin-associated cumulative sensory neuropathy is slowly reversible in most patients [9,10,17].

Among the various acute, transient neuropathic symptoms experienced by patients, cold sensitivity is the most common form, but it is rarely debilitating since patients can adapt to avoid cold stimuli. The temporary loss of awareness of breathing is relatively uncommon, but is probably the most alarming symptom. It is imperative that patients are warned about these symptoms and their transient nature prior to chemotherapy administration. Such patients can talk, and their breath exhalation is audible. Acute oxaliplatin-associated neurotoxicity can result in other disturbing symptoms such as visual field cuts, blurred vision and ptosis. Administration of FOLFOX regimens require a centrally implanted venous catheter to deliver outpatient infusional 5-FU. Since our study involved oral capecitabine, patients were not required to have central venous access. However, due to the occurrence of whole arm pain in the extremity used to infuse oxaliplatin, and due to extravasation injuries, we amended our protocol to recommend placement of central venous access devices.

There is no standard treatment for oxaliplatin-related neurotoxicity. A variety of strategies have been employed to prevent or treat oxaliplatin neurotoxicity [10,30-39], including carbamazepine, gabapentin, alpha lipoic acid, amifostine, glutathione, and celecoxib. The largest experience is the retrospective analysis of the benefit of infusing magnesium and calcium prior to oxaliplatin administration in 96 patients compared to 65 patients who did not receive magnesium/calcium. The percentage of patients with grade 3 distal paresthesia was lower in Ca/Mg group (7% vs 26%, p = 0.001), and acute symptoms such as distal and lingual paresthesia were much less frequent and severe in the Ca/Mg group. The authors concluded that Ca/Mg infusions seemed to reduce the incidence and intensity of acute oxaliplatin-induced symptoms and might delay cumulative neuropathy [32]. A large, randomized trial will be required to clarify the link between acute, transient symptoms and the likelihood of development of chronic sensory neuropathy, and confirm whether strategies such as Ca/Mg infusions reduce the neurotoxicity without impacting on the anti-tumor efficacy.

Another approach involves the administration of six cycles of FOLFOX-7, followed by the use of biweekly FULV2 for 12 cycles; oxaliplatin was then re-introduced for six cycles, or earlier if disease progression occurred on FULV2 [40]. The intention is to allow recovery from neurotoxicity during the oxaliplatin-free period which may allow the re-introduction of oxaliplatin. Preliminary results demonstrate that this is a convenient regimen with no detrimental effects on efficacy.

Several cases have been reported in the literature concerning peripheral neuropathy in association with 5-FU and capecitabine therapy. Stein reported two subjects who developed symptoms of pain and weakness in the lower extremities while receiving fluorouracil and levamisole [41]. EMG revealed axonal and demyelinting polyneuropathy involving small and large fibers. Two patients treated in a phase I trial of oral 5-FU, leucovorin and eniluracil, an inhibitor of dihydropyrimidine dehydrogenase, developed delayed onset symptoms of unsteady gait and reduced sensation in the legs [42]. EMG and NCS revealed sensorimotor polyneuropathy. Saif reported two patients who experienced either right leg weakness with foot drop or perioral and upper extremity paresthesias with capecitabine [43]. EMG and NCS showed sensorimotor peripheral neuropathy in both patients. In each of these three reports, other common etiologies of peripheral neuropathy were excluded. Couch et al reported a patient given capecitabine who experience acute onset of neuromuscular symptoms, the most prominent of which was trismus, [44]. Peripheral neuropathy may thus represent an extremely rare complication of fluorouracil or capecitabine therapy. The possible contribution of capecitabine to the neurologic symptoms in the patients in the current trial is unclear. However, the neurophysiologic studies obtained shortly after administration of oxaliplatin are unique and are reminiscent of neuromyotonia.

## Conclusion

The use of oxaliplatin chemotherapy is increasing and has resulted in significant improvements in outcomes in colorectal cancer. However, oxaliplatin-associated neurotoxicity can cause detrimental effects to the patient's quality of life and may require dose reduction or discontinuation. Our study provides detailed information on the incidence, type and duration of oxaliplatin neurotoxicity. Further understanding of oxaliplatin-associated neurotoxicity is necessary to warn patients of potential side effects and to facilitate strategies to prevent or treat this neurotoxicity. Optimizing the quality of life of cancer is of paramount importance. Continued research on oxaliplatin will help achieve this goal while also providing further progress with respect to clinical benefit outcomes.

## Abbreviations

5-FU, 5-fluorouracil; LV, leucovorin; FOLFOX, a combination of folinic acid (leucovorin), 5-FU and oxaliplatin given on a fortnightly schedule; FOLFIRI, a combination of folinic acid (leucovorin) given with 5-FU and irinotecan on a fortnightly schedule; NCI, National Cancer Institute; EMG, electromyogram; NCS, nerve conduction study

## Competing interests

The author(s) declare that they have no competing interests.

## Authors' contributions

GDL participated in the conduct of this trial and drafted the manuscript. MW participated in the conduct of this trial. MGQ participated in the conduct of this trial. SF participated in the conduct of this trial. NH participated in the conduct of this trial. BS participated in the conduct of this trial. RT participated in the conduct of this trial. JLG conceived of the study, coordinated its design and implementation, and wrote the final version. All authors read and approved the final manuscript.

## Pre-publication history

The pre-publication history for this paper can be accessed here:



## Figures and Tables

**Table 1:** Questionnaire documenting upper extremity symptoms of acute and chronic neuropathy experienced during or after oxaliplatin-based chemotherapy.

**Upper Extremity Symptoms**			If you had symptoms during the last cycle....									
			
			How much of the symptoms did you have?					Did the symptoms affect your daily activities?				
			
Do you have....			Hardly any → Very much					Hardly at all bothered → Extremely bothered				
Tingling (pins and needles)	Yes	No	1	2	3	4	5	1	2	3	4	5
Numbness	Yes	No	1	2	3	4	5	1	2	3	4	5
Difficulty telling the difference between rough and smooth surfaces	Yes	No	1	2	3	4	5	1	2	3	4	5
Difficulty feeling hot things	Yes	No	1	2	3	4	5	1	2	3	4	5
Difficulty feeling cold things	Yes	No	1	2	3	4	5	1	2	3	4	5
A greater than normal sense of touch (i.e. putting on gloves)	Yes	No	1	2	3	4	5	1	2	3	4	5
Burning pain or discomfort without cold	Yes	No	1	2	3	4	5	1	2	3	4	5
Burning pain or discomfort with cold	Yes	No	1	2	3	4	5	1	2	3	4	5
Difficulty identifying objects in your hand (i.e. coin)	Yes	No	1	2	3	4	5	1	2	3	4	5
Do you have involuntary hand movements	Yes	No	1	2	3	4	5	1	2	3	4	5

**Table 2:** Questionnaire documenting lower extremity symptoms of acute and chronic neuropathy experienced during or after oxaliplatin-based chemotherapy.

**Lower Extremity Symptoms**			If you had symptoms during the last cycle....									
			
			How much of the symptoms did you have?					Did the symptoms affect your daily activities?				
			
Do you have....			Hardly any → Very much					Hardly at all bothered → Extremely bothered				
Tingling (pins and needles)	Yes	No	1	2	3	4	5	1	2	3	4	5
Numbness	Yes	No	1	2	3	4	5	1	2	3	4	5
Difficulty telling the difference between rough and smooth surfaces	Yes	No	1	2	3	4	5	1	2	3	4	5
Difficulty feeling hot things	Yes	No	1	2	3	4	5	1	2	3	4	5
Difficulty feeling cold things	Yes	No	1	2	3	4	5	1	2	3	4	5
A greater than normal sense of touch (i.e. discomfort with socks)	Yes	No	1	2	3	4	5	1	2	3	4	5
Burning pain or discomfort without cold	Yes	No	1	2	3	4	5	1	2	3	4	5
Burning pain or discomfort with cold	Yes	No	1	2	3	4	5	1	2	3	4	5
Legs feel heavy	Yes	No	1	2	3	4	5	1	2	3	4	5

**Table 3:** Questionnaire documenting oral or facial symptoms of acute and chronic neuropathy experienced during or after oxaliplatin-based chemotherapy.

**Oral/Facial Symptoms**			If you had symptoms during the last cycle....									
			
			How much of the symptoms did you have?					Did the symptoms affect your daily activities?				
			
Do you have....			Hardly any → Very much					Hardly at all bothered → Extremely bothered				
Jaw pain	Yes	No	1	2	3	4	5	1	2	3	4	5
Eyelids drooping	Yes	No	1	2	3	4	5	1	2	3	4	5
Throat discomfort	Yes	No	1	2	3	4	5	1	2	3	4	5
Ear pain	Yes	No	1	2	3	4	5	1	2	3	4	5
Tingling in mouth	Yes	No	1	2	3	4	5	1	2	3	4	5
Difficulty with speech	Yes	No	1	2	3	4	5	1	2	3	4	5
Burning or discomfort of your eyes	Yes	No	1	2	3	4	5	1	2	3	4	5
Loss of any vision	Yes	No	1	2	3	4	5	1	2	3	4	5
Feeling shock/pain down back	Yes	No	1	2	3	4	5	1	2	3	4	5
Problems with breathing	Yes	No	1	2	3	4	5	1	2	3	4	5

**Table 4:** Worst grade of toxicity across all cycles of therapy

	No toxicity	Grade 1	Grade 2	Grade 3	Grade 4
Dyesthesia	12.8%	70.9%	11.6%	4.7%	0%
Paresthesia	5.7%	66.3%	19.8%	7%	1.2%

**Table 5:** Duration of dysesthesia and paresthesias (21 day cycles)

**Dysesthesias**			
Cycle number	Median duration (days)	Percent persisting ≥ 14 days	Percent persisting ≥ 21 days

1	5	9.5%	3.2%
3	7	14.3%	6.1%
6	10.5	38.2%	17.6%
9	13.5	50%	37.5%
12	21	60%	60%

**Paresthesias**			

Cycle number	Median duration (days)	Percent persisting ≥ 14 days	Percent persisting ≥ 21 days

1	7	19.7%	6.1%
3	12.5	35.6%	18.6%
6	14	54.3%	37%
9	= 21	86.4%	77.3%
12	= 21	83.3%	83.3%

**Table 6:** Cumulative chronic sensory neuropathy rates in selected phase III trials involving oxaliplatin and 5-FU based combination chemotherapy

Ref.	Regimen	Oxaliplatin Dose: mg/m^2^	Planned Oxaliplatin Dose intensity: mg/m^2^/wk	Treatment	≥ grade 3
25	OX + FU/LV^a ^chronotherapy	Both arms: 20 days 1–5 q 3 wk 20/10.5 hr CI; peak at 4:00 pm	33.3	First line	16%
	Constant rate	20/24 hr CI			31%
26	OX + FU/LV^b^	125/6 hr day 1 q 3 wk 10:00 am to 4:00 pm	41.7	First line	10%
3	FOLFOX 4	85/2 hr day 1 q 2 wk	42.5	First line	18.2%
4	FOLFOX 4	85/2 hr day 1 q 2 wk	42.5	First line	18%
	IROX	85/1.5 hr day 1 q 3 wk	28.3		7%
5	FOLFOX 6	100/2 hr day 1 q 2 wk	50	First line	34%
27	FUFOX	50/2 hr days 1, 8, 15, 22 q 5 wk^c^	40	First line	17%
28	FUFOX	50/x hr days 1, 8, 15, 22 q 5 wk^c^	40	First line	25%
	CAPOX	70/x hr d 1, 8 q 3 wk	46.7		16%
17	FOLFOX 4	85/2 hr day 1 q 2 wk	42.5	Adjuvant	12%
5	FOLFOX 6	100/2 hr day 1 q 2 wk	50	Second line^d^	20%
29	FOLFOX 4	85/2 hr day 1 q 2 wk	42.5	Second or third line^e^	3%^f^

FOLFOX: oxaliplatin and biweekly FULV2; IROX: oxaliplatin and irinotecan;

FUFOX: oxaliplatin and weekly 24 hour infusional 5-FU/LV; CAPOX: capecitabine and oxaliplatin

^a ^5-FU 600 mg/m^2 ^and LV 300 mg/m^2 ^daily for 5 days over 12 hr with peak at either 04:00 am or over 24 hr by constant rate infusion; ^b ^5-FU 700 mg/m^2 ^and LV 300 mg/m^2 ^daily for 5 days over 12 hr with peak at 04:00 am; ^c ^after the first four cycles, oxaliplatin given every 2 weeks; ^d ^patients had previously progressed on FOLFIRI; ^e ^Grade 3–4 acute, cold-sensitive paresthesia also occurred in 3% of patients.
